# Calcineurin Inhibitor-Induced and Ras-Mediated Overexpression of VEGF in Renal Cancer Cells Involves mTOR through the Regulation of PRAS40

**DOI:** 10.1371/journal.pone.0023919

**Published:** 2011-08-23

**Authors:** Aninda Basu, Pallavi Banerjee, Alan G. Contreras, Evelyn Flynn, Soumitro Pal

**Affiliations:** 1 Division of Nephrology and Transplantation Research Center, Children's Hospital, Boston, Massachusetts, United States of America; 2 Department of Pediatrics, Harvard Medical School, Boston, Massachusetts, United States of America; Ohio State University, United States of America

## Abstract

Malignancy is a major problem in patients treated with immunosuppressive agents. We have demonstrated that treatment with calcineurin inhibitors (CNIs) can induce the activation of proto-oncogenic Ras, and may promote a rapid progression of human renal cancer through the overexpression of vascular endothelial growth factor (VEGF). Interestingly, we found that CNI-induced VEGF overexpression and cancer cell proliferation was inhibited by rapamycin treatment, indicating potential involvement of the mammalian target of rapamycin (mTOR) pathway in this tumorigenic process. Here, we examined the role of mTOR pathway in mediating CNI- and Ras-induced overexpression of VEGF in human renal cancer cells (786-0 and Caki-1). We found that the knockdown of raptor (using siRNA) significantly decreased CNI-induced VEGF promoter activity as observed by promoter-luciferase assay, suggesting the role of mTOR complex1 (mTORC1) in CNI-induced VEGF transcription. It is known that mTOR becomes activated following phosphorylation of its negative regulator PRAS40, which is a part of mTORC1. We observed that CNI treatment and activation of H-Ras (through transfection of an active H-Ras plasmid) markedly increased the phosphorylation of PRAS40, and the transfection of cells using a dominant-negative plasmid of Ras, significantly decreased PRAS40 phosphorylation. Protein kinase C (PKC)-ζ and PKC-δ, which are critical intermediary signaling molecules for CNI-induced tumorigenic pathway, formed complex with PRAS40; and we found that the CNI treatment increased the complex formation between PRAS40 and PKC, particularly (PKC)-ζ. Inhibition of PKC activity using pharmacological inhibitor markedly decreased H-Ras-induced phosphorylation of PRAS40. The overexpression of PRAS40 in renal cancer cells significantly down-regulated CNI- and H-Ras-induced VEGF transcriptional activation. Finally, it was observed that CNI treatment increased the expression of phosho-PRAS40 in renal tumor tissues *in vivo*. Together, the phosphorylation of PRAS40 is critical for the activation of mTOR in CNI-induced VEGF overexpression and renal cancer progression.

## Introduction

Recent improvements in immunosuppressive therapies have significantly reduced the incidence of acute rejection of allografts, and increased the survival of transplant patients [1], [2]. However, these agents may also contribute to higher rates of mortality due to an increased risk of cancer [3], [4], [5], [6]. It has been established that cancer represents the second main cause of death in renal transplant patients with normal function of the graft [7]. It has also been shown that the transplant environment can accelerate recurrence or progression of cancer [3], . Thus, therapeutic targets need to be developed in order to prevent cancer development in patients under immunosuppressive therapy.

The immunosuppressive agents are thought to compromise immune surveillance mechanism(s) of tumor cells and/or interfere with normal DNA repair mechanisms [4], [5], [8]. In particular, calcineurin inhibitors (CNIs) are excellent immunosuppressive agents to inhibit allograft rejection; however they may promote the growth of different tumors [9], [10], [11], [12]. The calcineurin complex consists of three subunits, the catalytic A, the regulatory B, and calmodulin [13]. The cellular calcium activates the catalytic subunit for its function as serine/threonine phosphatase, resulting in the activation of the nuclear factor of activated T cells (NFAT) family of transcription factors [14]. The CNI cyclosporine (CsA) binds to cyclophylin, a cytoplasmic protein, and the resultant complex binds to the regulatory B subunit of calcineurin and prevents the activation of NFAT [15]. However, apart from inhibiting NFAT, the CNIs may also regulate other signaling molecules playing important roles in tumor growth [16], [17]. Hojo *et al.* [9] showed that CsA promotes cancer progression and metastasis by direct cellular effect(s) through transforming growth factor-β (TGF-β) production, which is independent of its effect on the immune system of the host. Koehl *et al.* [18] reported that CsA treatment promotes the development of post-transplantation cancer, which is highly dependent on the process of tumor angiogenesis. Similarly, Guba *et al.* [19] suggested that CsA treatment can induce the expression of angiogenic cytokines.

Vascular endothelial growth factor (VEGF) is one of the most potent angiogenic cytokines that plays important role in tumor growth [20], [21]. We have recently demonstrated that the treatment with CNIs induces overexpression of VEGF, and promotes a rapid progression of human renal cancer [22]. CNI-induced VEGF overexpression is regulated at both transcriptional and post-transcriptional level [22], [23]. We have also found that CNIs can activate the proto-oncogenic H-Ras in human renal cancer cells [24]; and we have shown that CNI-induced VEGF overexpression is mediated through the activation of protein kinase C (PKC)-ζ and PKC-δ [22], [23], which are potential downstream targets of Ras [25].

In contrast to CNIs, the mammalian target of rapamycin (mTOR) inhibitor rapamycin (RAPA) may have a completely opposite effect in terms of tumor development [10], [19], [26]. The transplant patients receiving RAPA treatment do not develop cancer at the same rate as those receiving other immunosuppressive agents such as CNIs [27], [28]. It has been shown that RAPA treatment may have an anti-angiogenic effect [19]. Interestingly, we have recently demonstrated that RAPA treatment can significantly inhibit CNI-induced VEGF mRNA stability [23], and CNI-induced proliferation of human renal cancer cells [24]. These results clearly suggest a possible role of mTOR in CNI-induced tumorigenic pathways. In support of these observations, it has been reported that the Akt-mTOR pathway is required for CNI-induced tumor growth [29]. In addition, both PKC-ζ and PKC-δ may activate the Akt-mTOR pathway [30], [31], [32], [33].

The mTOR pathway plays a key role in cell survival, growth, protein synthesis, cellular metabolism, and angiogenesis [34], [35]. Alterations in the pathway regulating mTOR occur in many solid malignancies, including kidney cancer [36], [37], [38]. mTOR, which is constitutively activated in many cancers by deregulated activation of oncogenes or loss of tumor suppressor genes, functions as macromolecular complexes [39]. The mTOR complex1 (mTORC1), containing raptor, is RAPA sensitive; while the mTOR complex2 (mTORC2), containing rictor, is RAPA insensitive [34], [39]. It has recently been established that a proline-rich Akt substrate of 40 kDa (PRAS40) can negatively regulate mTOR activity [40], [41]. Before getting phosphorylated by Akt, PRAS40 binds to raptor and sequesters raptor from mTORC1; this leads to the disruption of mTORC1 similar to the effect of RAPA [40], [42]. The interaction of PRAS40 with raptor competes with the interaction of raptor with S6K1 and 4E-BP1 [42], [43]. In addition, this interaction of PRAS40 is very specific for the mTORC1, as PRAS40 does not associate with or disrupt mTORC2 [34].

In this study, we show that CNI-induced and Ras-PKC-mediated VEGF overexpression can be channeled through the mTORC1 signaling pathway, and this is mediated through the regulation of PRAS40. We demonstrate that CNI treatment and activation of H-Ras and PKC can lead to the phosphorylation of PRAS40; and overexpression of PRAS40 leads to the down-regulation of CNI- and Ras-induced VEGF transcriptional activation. The results of our study suggest a novel cross-talk among Ras, PKC and mTOR in regulating CNI-induced VEGF overexpression.

## Results

### Involvement of the mTOR complex1 in Calcineurin Inhibitor-Induced VEGF Overexpression in Human Renal Cancer Cells

**Figure 1:**
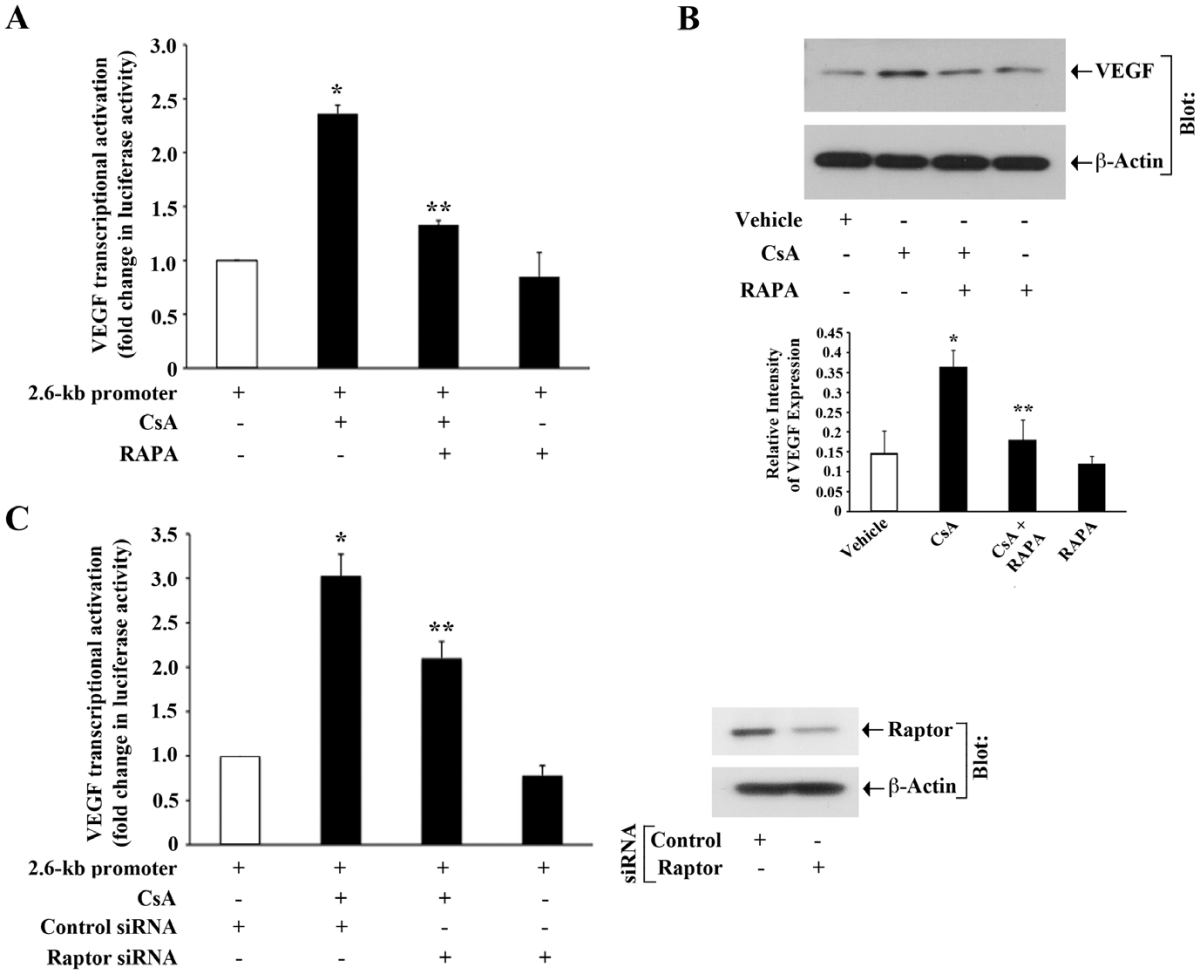
Role of mTORC1 in CNI-induced VEGF transcriptional activation. *A,* 786-0 cells were transfected with the 2.6-kb VEGF promoter-luciferase construct (0.5 µg/well). After transfection, the cells were cultured for 12 hour, and then treated overnight (12 hour) with different combinations of CsA (5.0 µg/ml) and RAPA (10.0 ng/ml) or vehicle alone (control). Following 24 hour of transfection, the cells were harvested, and fold change in luciferase activity was calculated as the relative luciferase counts from each group of cells compared with that of cells treated with vehicle alone. The data reflect three independent experiments. *Columns,* average of triplicate readings of two different samples; *error bars,* SD. *B,* 786-0 cells were treated with different combinations of CsA (5.0 µg/ml) and RAPA (10.0 ng/ml) or vehicle alone (control) for 24 hour. Whole cell lysates were prepared, and Western blot analysis was performed using anti-VEGF and anti-β-actin to quantitate the protein expression of VEGF and β-actin respectively. The bar graph below the Western blot illustrates the relative expression of VEGF by densitometry, wherein the signals were standardized to the expression of the internal control β-actin. Representative of three independent experiments. *Columns,* average of relative intensity of VEGF expression from three different blots; *bars,* SD. *C,* 786-0 cells were transfected with either raptor siRNA (25 nM) or control siRNA. Following 24 hour of siRNA transfection, cells were transfected with the 2.6-kb VEGF promoter-luciferase construct (0.5 µg/well). After 12 hour of plasmid transfection, the cells were treated overnight (12 hour) with either CsA (5.0 µg/ml) or vehicle alone (control). The cells were harvested, and fold change in luciferase activity was calculated as the relative luciferase counts from each group of cells compared with that of cells transfected with control siRNA and treated with vehicle alone. The data reflect two independent experiments. *Columns,* average of triplicate readings of samples; *error bars,* SD. The knockdown of raptor was confirmed by Western blot analysis after 48 hour of siRNA transfection (*right panel).* (A–B) *, *p*<0.01 compared with vehicle-treated cells; **, *p*<0.01 compared with only CsA-treated cells. (C) *, *p*<0.01 compared with control siRNA-transfected and vehicle-treated cells; **, *p*<0.05 compared with control siRNA-transfected and CsA-treated cells.

We have recently demonstrated that treatment with calcineurin inhibitors (CNIs) can promote VEGF overexpression in human renal cancer cells through both transcriptional and post-transcriptional regulations [22], [23]. We have also shown that CNI-induced VEGF mRNA stability, and CNI-induced renal cancer cell proliferation are markedly inhibited following treatment with the mTOR inhibitor rapamycin (RAPA) [23], [24]. Our findings clearly indicated the possible role of mTOR in CNI-induced tumorigenic pathways. Here, we first examined the role of mTOR in CNI-induced VEGF transcriptional activation in human renal cancer cells (786-0 and Caki-1). Cells were transfected with the VEGF promoter-luciferase plasmid, and then treated with the CNI cyclosporine (CsA) in absence or presence of RAPA. As shown in Figure 1A, CsA treatment promoted VEGF transcriptional activation in 786-0 cells, and RAPA treatment significantly inhibited CsA-induced VEGF promoter activity. We found a similar result (data not shown) in Caki-1 cells. Next, we also observed that CsA treatment induced VEGF protein expression as observed by Western blot analysis; and the treatment with RAPA significantly inhibited CsA-induced VEGF expression (Figure 1B).

We next examined the role of mTOR complex1 (mTORC1) in CNI-induced VEGF transcription. As discussed earlier, raptor is a part of mTORC1 [34], [39]. Here, we observed that the knockdown of raptor using siRNA significantly downregulated CNI-induced VEGF promoter activity (Figure 1C). The knockdown of raptor (∼70%) was confirmed by Western blot analysis (Figure 1C, *right panel*). Together, these observations clearly suggest the involvement of mTORC1 in CNI-induced VEGF overexpression in renal cancer cells.

### Treatment with CNI, and the Activation of H-Ras Promotes Phosphorylation of PRAS40

**Figure 2:**
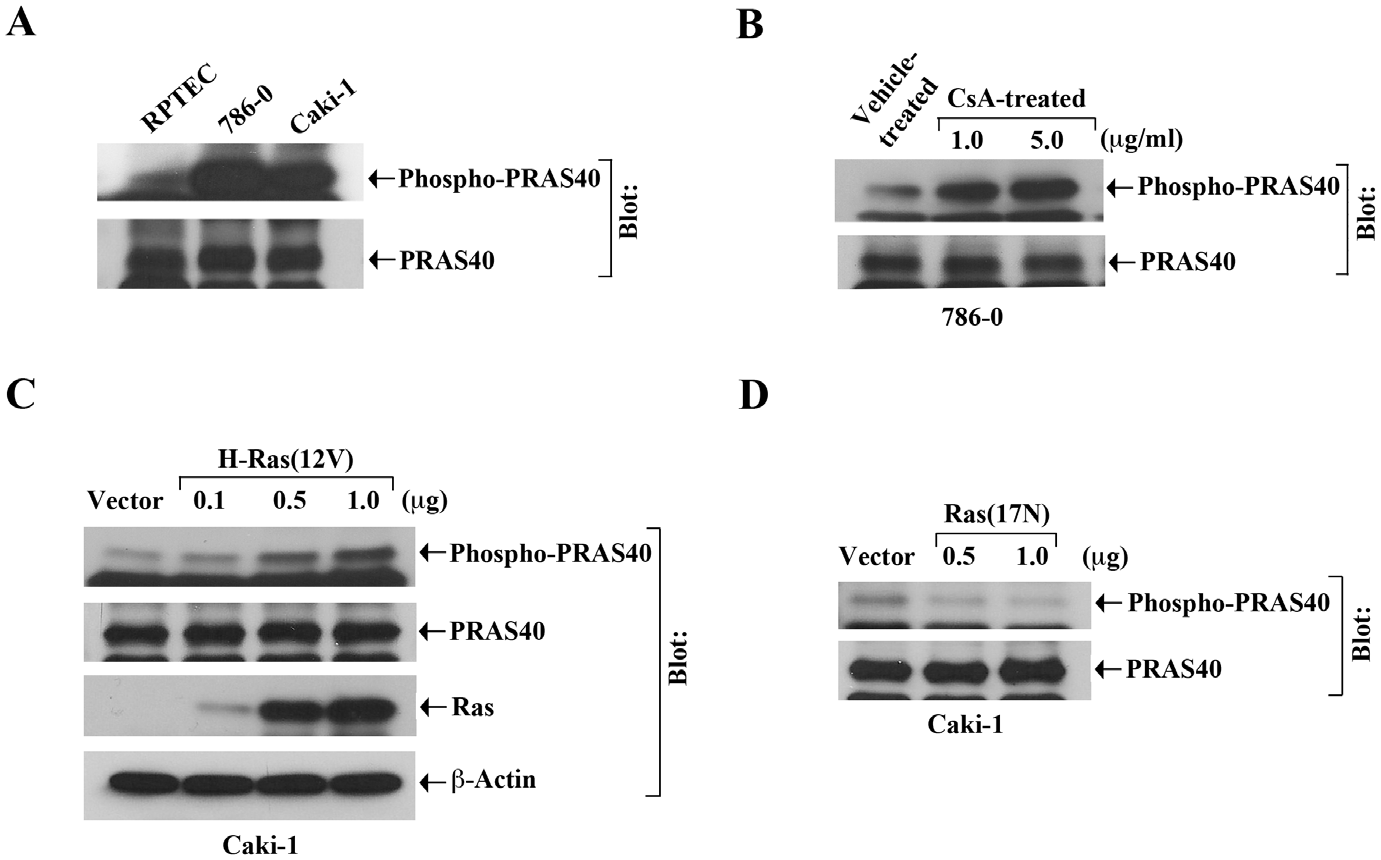
CNI treatment and H-Ras activation increases the phosphorylation of PRAS40. *A,* The expression of phospho-PRAS40 and PRAS40 was measured in whole cell lysates of RPTEC, 786-0, and Caki-1 by Western blot analysis using anti-phospho-PRAS40 and anti-PRAS40. *B,* 786-0 cells were treated with different concentrations (1.0 and 5.0 µg/ml) of CsA or with vehicle alone (control) for 3 hour. Cells were lysed, and the expression of phospho-PRAS40 and PRAS40 was measured by Western blot analysis. *C,* Caki-1 cells were transfected with either increasing concentrations (0.1–1.0 µg/well) of H-Ras(12V) or empty expression vector (control) for 24 hour. Cells were lysed, and the expression of phospho-PRAS40, PRAS40, Ras, and β-actin in cell lysates was measured by Western blot analysis. *D,* Caki-1 cells were transfected with either different concentrations (0.5 and 1.0 µg/well) of the dominant-negative Ras(17N) or empty expression vector (control) for 24 hour. Cells were lysed, and the expression of phospho-PRAS40, and PRAS40 was measured by Western blot analysis. (A–D) Representative of three independent experiments with similar findings.

We have recently shown that CNI treatment can induce activation of H-Ras in renal cancer cells [24]. In a recent report [44], it has been demonstrated that the activation of Ras can promote mTOR signaling. As discussed earlier, PRAS40 acts as a negative regulator of mTORC1, and inhibits its activity for the downstream signaling events [34], [40], [41]. After phosphorylation, PRAS40 gets disassociated from raptor of mTORC1, and thus mTOR becomes activated. As our earlier experiment indicated the involvement of raptor in CNI-induced VEGF transcription, here we wished to evaluate if CNI treatment and activation of H-Ras could regulate PRAS40 phosphorylation. First, we checked the expression of phospho-PRAS40 in normal renal epithelial cells (RPTEC), and in 786-0 and Caki-1 renal cancer cells. Through Western blot analysis, we found that the expression of phospho-PRAS40 was markedly higher in 786-0 and Caki-1 cells compared with RPTEC (Figure 2A, *upper panel*); however, there was no significant change in the expression of total PRAS40 in these cells (Figure 2A, *lower panel*). This observation suggests that the mTOR pathway is active in renal cancer cells.

We next determined the effect of CsA treatment on PRAS40 phosphorylation. 786-0 cells were treated with either increasing concentrations of CsA or the vehicle alone; and the expression of phospho-PRAS40 was examined by Western blot analysis. As shown in Figure 2B, CsA treatment markedly increased the expression level of phospho-PRAS40 compared with vehicle-treated control (*upper panel*); however, there was no significant change in the expression of total PRAS40 following CsA treatment (*lower panel*).

Next, we sought to evaluate the effect of H-Ras activation on PRAS40 phosphorylation. To this end, Caki-1 cells were transfected with either increasing concentrations of the plasmid expressing activated form of H-Ras, H-Ras(12V), or the empty expression vector. Following transfection, the expression of phospho-PRAS40 and total PRAS40 was measured. We found that the activation of H-Ras significantly increased the level of phosho-PRAS40 compared with vector-transfected control (Figure 2C, *first panel*); there was no significant change in total PRAS40 following H-Ras activation (Figure 2C, *second panel*). The overexpression of H-Ras(12V) in these cells was confirmed by Western blot analysis (Figure 2C, *third panel*).

Finally, we checked the effect of the inhibition of endogenous Ras on PRAS40 phosphorylation. The Caki-1 cells were transfected either with the dominant-negative mutant of Ras, Ras(17N), or the empty vector, and the expression of phospho-PRAS40 was measured. As shown in Figure 2D, inhibition of Ras decreased the expression of phospho-PRAS40 (*upper panel*); however, there was no significant change in the expression of total PRAS40 (*lower panel*). Together, these observations suggest that CNI treatment and activation of the H-Ras pathway in human renal cancer cells can induce mTOR through increased phosphorylation of PRAS40.

### PKC Forms Complex with PRAS40, and can Induce Its Phosphorylation

**Figure 3:**
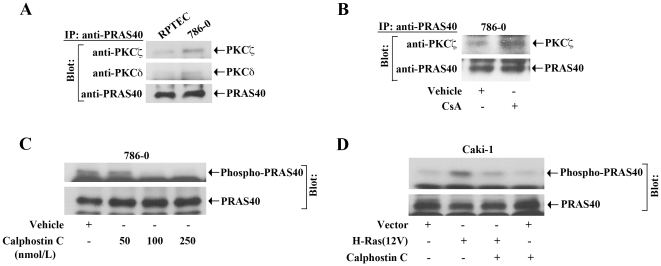
PKC forms complex with PRAS40, and can promote its phosphorylation. *A,* Lysates of RPTEC and 786-0 cells were immunoprecipitated with anti-PRAS40. *B,* 786-0 cells were treated with either CsA (5.0 µg/ml) or vehicle alone (control) for 2 hour. Cell lysates were immunoprecipitated with anti-PRAS40. (A–B) Immunoprecipitates (IP) were captured by protein A-Sepharose beads, boiled in SDS buffer, and separated by SDS-PAGE. Western blot analysis was performed using either anti-PKCζ, or anti-PKCδ, or anti-PRAS40. *C,* 786-0 cells were treated with either increasing concentrations (50–250 nmol/L) of calphostin C or vehicle alone (control) for 3 hour. Cells were lysed, and the expression of phospho-PRAS40 and PRAS40 was measured by Western blot analysis. *D,* Caki-1 cells were pretreated with either calphostin C (100 nmol/L) or vehicle alone; and cells were then transfected with either H-Ras(12V) (1.0 µg/well) or vector alone for 24 hour, in absence or presence of calphostin C. Cells were lysed, and the expression of phospho-PRAS40 and PRAS40 was measured by Western blot analysis. (A–D) Representative of three independent experiments with similar findings.

In our previous report [22], we have demonstrated that both PKC-ζ and PKC-δ are critical intermediary signaling molecules for CNI-induced VEGF transcriptional activation; and these PKC isoforms may serve as potential downstream targets of active Ras [25]. Here, we sought to determine if PKC could regulate the phosphorylation of PRAS40. First, we checked whether there was any complex formation between PRAS40 and either PKC-ζ and PKC-δ in RPTEC and 786-0 cells under basal condition. By immunoprecipitation, we observed that indeed both PKC-ζ and PKC-δ could make complex with PRAS40 (Figure 3A); however, intensity of the complex was much stronger in cancer cells versus normal renal epithelial cells. We next examined if CNI treatment could modulate the complex formation between PRAS40 and PKC-ζ and PKC-δ in 786-0 cells. As shown in Figure 3B, the treatment with CsA markedly increased the complex between PRAS40 and PKC-ζ compared with vehicle-treated control; however, there was no significant change (data not shown) in the complex formation between PRAS40 and PKC-δ.

Next, we tested if inhibition of PKC through the treatment of pharmacological inhibitor could decrease the phosphorylation of PRAS40. 786-0 cells were treated with either increasing concentrations of calphostin C or the vehicle alone. Following treatment, the expression of phospho-PRAS40 and total PRAS40 was measured by Western blot analysis. As shown in Figure 3C, the treatment with calphostin C markedly decreased the level of phosho-PRAS40 compared with vehicle-treated control (*upper panel*); however, there was no significant change in the expression of total PRAS40 (*lower panel*). Together, these observations suggest that PKC can associate with PRAS40, and regulate its phosphorylation. However, it cannot be concluded if there is a direct complex formation between PKC and PRAS40, and whether some other associated molecules are involved in PKC-mediated PRAS40 phosphorylation.

### Inhibition of PKC Down-Regulates H-Ras-Induced Phosphorylation of PRAS40

In our earlier experiments, we have demonstrated that H-Ras activation could induce PRAS40 phosphorylation. Here, we wished to explore if the inhibition of PKC could down-regulate H-Ras-induced phosphorylation of PRAS40 in renal cancer cells. To this end, Caki-1 cells were transfected with either H-Ras(12V) or the empty expression vector in absence or presence of the PKC inhibitor calphostin C. Following transfection, the expression of phospho-PRAS40 and total PRAS40 was measured. As shown in Figure 3D (*upper panel*), activation of H-Ras induced the phosphorylation of PRAS40 compared with vector-transfected control; and the inhibition of PKC significantly down-regulated H-Ras-induced PRAS40 phosphorylation. There was no significant change in the expression of total PRAS40 following H-Ras activation and PKC inhibition (Figure 3D, *lower panel)*. These observations clearly suggest that the Ras-PKC pathway, which is critical for CNI-induced tumorigenic signaling events, can phosphorylate PRAS40, and may thus activate mTOR.

### Overexpression of PRAS40 Inhibits CNI- and H-Ras-Induced Transcriptional Activation of VEGF

**Figure 4:**
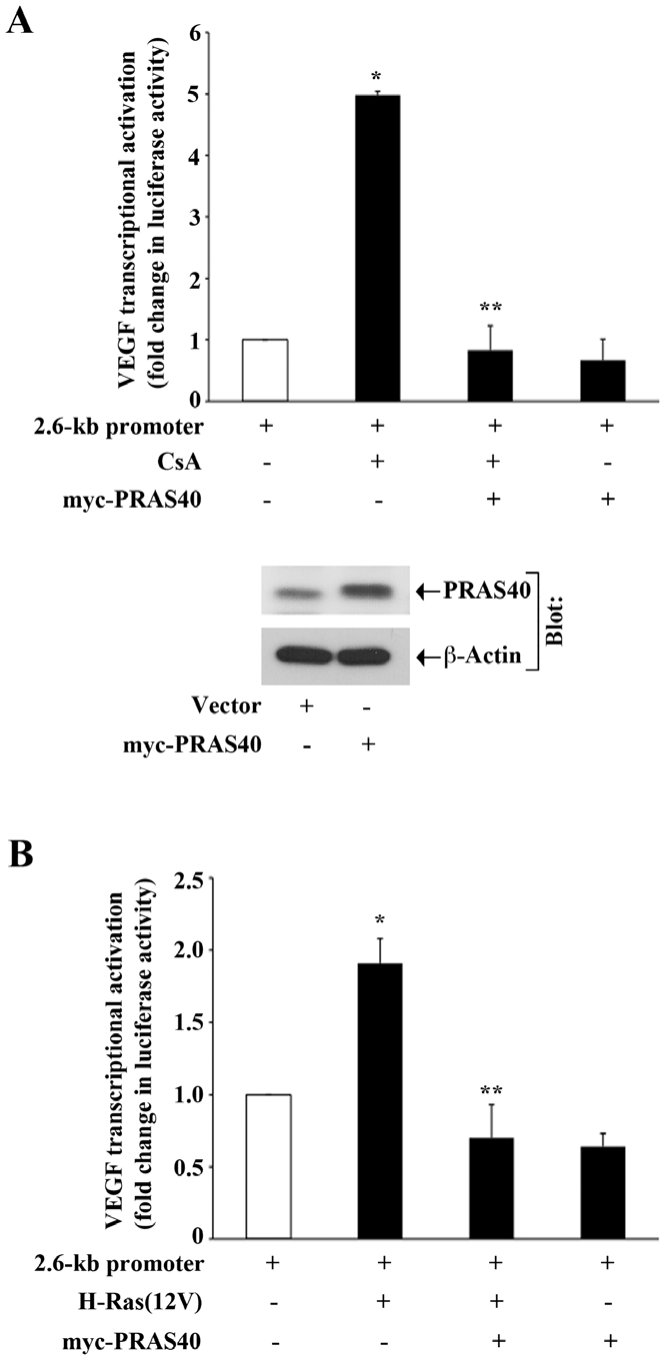
Overexpression of PRAS40 inhibits CNI- and Ras-induced VEGF transcriptional activation. *A, top,* 786-0 cells were co-transfected with the 2.6-kb VEGF promoter-luciferase construct (0.5 µg/well) and either a PRAS40 overexpression plasmid (myc-PRAS40) (0.5 µg/well) or empty vector. After transfection, cells were cultured for 12 hour, and then treated overnight (12 hour) with either CsA (5.0 µg/ml) or vehicle alone (control). Following CsA treatment, cells were harvested, and fold change in luciferase activity was calculated as the relative luciferase counts from each group of cells compared with that of cells transfected with empty vector and treated with vehicle alone. *A, bottom,* The overexpression of myc-PRAS40 plasmid in transfected cells was confirmed by Western blot analysis using anti-PRAS40; and the expression of β-actin was measured as internal control. *B,* Caki-1 cells were co-transfected with the 2.6-kb VEGF promoter-luciferase construct (0.5 µg/well) and different combinations of H-Ras(12V), myc-PRAS40 and the empty vector (0.5 µg/well of each plasmid). Following 24 hour of transfection, the cells were harvested, and fold change in luciferase activity was calculated as the relative luciferase counts from each group of cells compared with that of cells transfected with empty vector. (A–B) The data reflect three independent experiments. *Columns,* average of triplicate readings of two different samples; *error bars,* SD. In A, *, *p*<0.01 compared with empty vector-transfected and vehicle-treated cells; **, *p*<0.01 compared with empty vector-transfected and CsA-treated cells. In B, * *p*<0.01 compared with vector-transfected cells; **, *p*<0.01 compared with vector- and H-Ras(12V)-transfected cells.

Our previous experiments suggested that CNI-induced and Ras-mediated signaling pathways could inactivate PRAS40 through its increased phosphorylation. Here, we first examined if the overexpression of PRAS40 could inhibit CNI-induced overexpression of VEGF in renal cancer cells. 786-0 cells were co-transfected with the VEGF promoter-luciferase construct and either PRAS40 overexpression plasmid or the empty expression vector. Cells were treated with either CsA or the vehicle alone. As shown in Figure 4A, CsA treatment increased VEGF transcriptional activation compared with vehicle-treated control; and the overexpression of PRAS40 significantly reduced CsA-induced VEGF promoter activity. The overexpression of PRAS40 in transfected cells was confirmed by Western blot analysis (Figure 4A, *lower panel*).

Next, we determined if the overexpression of PRAS40 could inhibit H-Ras-induced VEGF transcription. Caki-1 cells were co-transfected with the VEGF promoter-luciferase construct and H-Ras(12V) in absence or presence of the PRAS40 overexpression plasmid. Control cells were transfected with empty expression vectors. As shown in Figure 4B, activation of H-Ras increased VEGF transcriptional activation compared with vector-transfected cells; and the overexpression of PRAS40 significantly decreased H-Ras-induced VEGF promoter activity. Together, these findings suggest that CNI- and Ras-induced signaling events can promote VEGF transcriptional activation in an mTOR-dependent pathway through the regulation of PRAS40.

### CNI Treatment Increases the Phosphorylation of PRAS40 in Renal Tumor Tissues *in Vivo*

**Figure 5:**
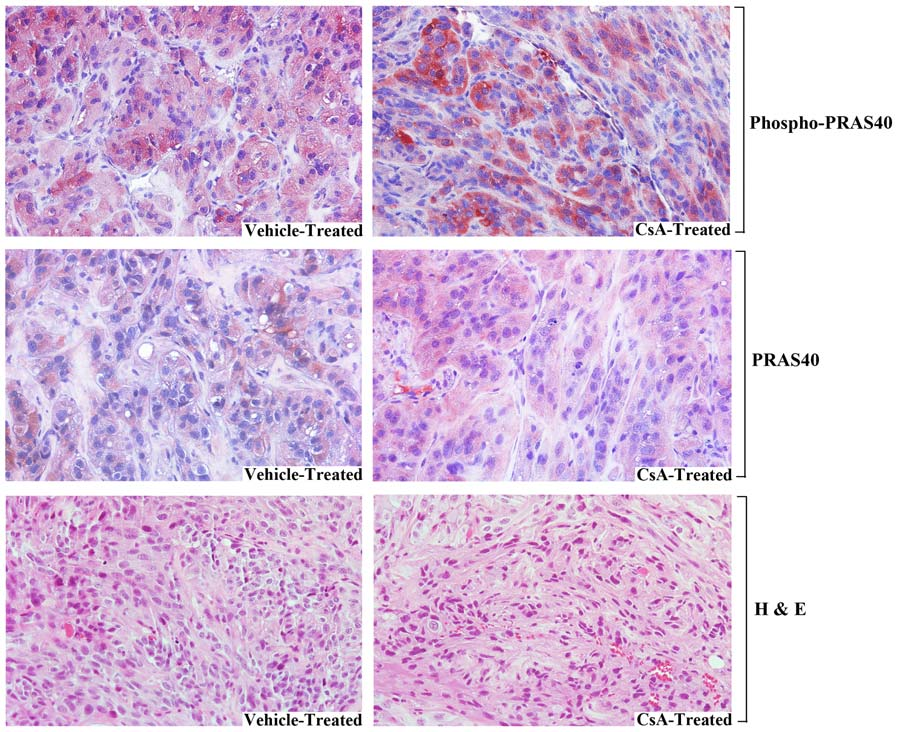
Treatment with CNI promotes the phosphorylation of PRAS40 in renal tumor tissues *in vivo*. Human renal cancer cells (1.0×10^6^; 786-0) were injected s.c. in nude (*nu/nu*) mice (*n* = 5 in each group), and they were treated either with CsA (10 mg/kg/day) or with the vehicle as control. Tumors were harvested at day 25 following tumor injection. Representative photomicrographs illustrate the immunohistochemical expression of phospho-PRAS40 (*top panels*) and PRAS40 (*middle panels*) in harvested renal tumor tissues (magnification X400). *Patches of dark red color,* expression of phosho-PRAS40, which was markedly increased in tumor tissues from CsA-treated mice. H & E, hematoxylin and eosin. Representative of three different tissue samples of both CsA- and vehicle-treated groups.

We have recently demonstrated that in immunodeficient (*nu/nu*) mice, CNI (CsA) treatment significantly accelerated the growth of human renal tumors (786-0) through VEGF-induced angiogenesis, compared with vehicle-treated controls [22]. However, we did not evaluate the expression level of phospho-PRAS40 in the tumors. Thus, here we examined the status of phospho-PRAS40 in these tumor tissues from CsA-treated as well as control mice. As shown in Figure 5, the expression of phospho-PRAS40 was markedly increased (as observed by patches of dark red staining) in renal tumor tissues obtained from CsA-treated mice (*top right panel*), compared with tumor tissues from the vehicle-treated control group (*top left panel*). However, there was no significant change in the expression of total PRAS40 in tumor tissues obtained from CsA-treated (*middle right panel*) or vehicle-treated group (*middle left panel*). Our *in vivo* data is similar to our *in vitro* findings, and it suggests that CNI-mediated and VEGF-induced accelerated growth of human renal tumors may involve increased phosphorylation of PRAS40, which may lead to the activation of mTOR pathway.

## Discussion

The development as well as rapid progression of cancer is a major problem in patients treated with immunosuppressive agents [3], [4], [5]. We have recently demonstrated that calcineurin inhibitors (CNIs) can promote rapid progression of human renal cancer through the overexpression of VEGF [22], [23]; and H-Ras and PKC may act as critical intermediary signaling molecules for CNI-induced VEGF overexpression [22], [24]. In this study, we show a novel pathway, in which CNI-induced and Ras-PKC-mediated signals can involve mTORC1 through the regulation of its inhibitory molecule PRAS40, and promote VEGF overexpression.

As discussed earlier, CNIs mediate their immunosuppressive function through inhibition of the calcineurin-NFAT pathway [15]. However, CNIs may also regulate other signaling molecules involved in the expression of VEGF and other genes [16], [17]. We have recently shown that CNI treatment can activate H-Ras, and can also induce the phosphorylation of its downstream targets, PKC-ζ and PKC-δ; and promotes the overexpression of VEGF in human renal cancer cells [22], [23]. Chen *et al.* [45] have reported that CsA-induced oxidative stress can up-regulate and activate PKC-ζ in virally infected human B cells, which may lead to the induction of lymphoproliferative disorders in transplant patients.

Interestingly, we have demonstrated that the CNI-induced VEGF overexpression and renal cancer cell proliferation is inhibited by RAPA treatment, suggesting the possible role of mTOR in CNI-induced tumorigenic pathways that involves Ras activation [23], [24]. In support to our observations, Carriere *et al.* [44] have recently reported that mitogenic and oncogenic activation of the Ras pathway can induce mTORC1; it has also been shown that the Akt-mTOR pathway is required for CNI-induced tumor growth [29]. In addition, both PKC-ζ and PKC-δ may promote induction of the Akt-mTOR pathway [30], [31], [32], [33]; and the PI-3K/Akt/mTOR-mediated signals can be channeled through HIF and Sp1 [46], [47], two major transcription factors for VEGF expression [25], [48].

In the present study, we find that raptor, which is a part of mTORC1 is critical for CNI-induced VEGF transcriptional activation. We show that CNI/Ras-induced and PKC-mediated overexpression of VEGF in human renal cancer cells involves PRAS40, a negative regulator of mTORC1 [34], [40], [41]. The CNI treatment as well as the activation of Ras and PKC promotes phosphorylation of PRAS40, which may lead to the induction of mTOR signaling pathway. Our study suggests the role of H-Ras in regulating PRAS40 phosphorylation; however, we cannot rule out the roles of other two Ras isoforms (K-Ras and N-Ras) in this process. Previously, we demonstrated that CNI treatment mediates a rapid progression of human renal tumor through VEGF-induced angiogenesis [22]; here, we show that the expression of phospho-PRAS40 is markedly increased in these renal tumor tissues following CNI treatment. The overexpression of PRAS40 significantly reduced CNI- and Ras-induced VEGF transcriptional activation. Thus, our study clearly suggests that mTOR is a critical signaling molecule in CNI-induced tumorigenic pathway(s) that may lead to VEGF overexpression in renal cancer. Although we find a major role of mTORC1, any possible involvement of mTORC2 in CNI-induced VEGF expression needs to be tested.

As discussed earlier, in contrast to CNIs, the mTOR inhibitor RAPA may have anti-angiogenic and anti-tumorigenic potential [10], [19]. It is a challenge for the clinicians to fix a safe but effective immunosuppressive agent for the treatment of transplant patients. It may be suggested that a combination therapy using both CNI (low dose) and RAPA treatment can be considered to achieve optimal immunosuppression, as well as to prevent cancer development/recurrence in transplant patients. In addition, our study defines a novel pathway, in which the overexpression of PRAS40 may limit CNI/Ras-induced and mTOR-mediated rapid progression of human renal cancer.

In summary, our study identifies that phosphorylation of PRAS40 may lead to the activation of mTOR signaling pathway in CNI-induced rapid progression of human renal cancer. The activation of H-Ras and PKC (possibly PKC-ζ) following CNI treatment can promote the phosphorylation of PRAS40, and thereby may relieve the inhibition of mTORC1. Thus, targeting this pro-tumorigenic pathway may serve as novel therapeutics for the prevention and treatment of renal cancer, particularly in CNI-treated patients.

## Materials and Methods

### Reagents

CsA (Novartis) was purchased from Children's Hospital Boston pharmacy, and RAPA was purchased from LC laboratories. The PKC inhibitor calphostin C was obtained from Calbiochem. The small interfering RNA (siRNA) for raptor and its control were purchased from Qiagen. The transfection of siRNA was performed using Lipofectamine 2000 (Invitrogen).

### Cell Lines

The human renal cancer cell lines (786-0 and Caki-1) were obtained from American Type Culture Collection. 786-0 cells were grown in RPMI 1640, and Caki-1 cells were grown in McCoy's medium supplemented with 10% fetal bovine serum (GIBCO). Human normal renal proximal tubular epithelial cells (RPTEC) were purchased from Clonetics and were grown in complete epithelial medium (REGM BulletKit).

### Plasmids

A 2.6-kb VEGF promoter-luciferase construct in pGL2 basic vector (Promega), containing full-length VEGF promoter sequence (−2361 to +298 bp relative to the transcription start site) was used in transient transfection assay [22], [48]. All the Ras mutant constructs were obtained as generous gifts from Roya Khosravi-Far (Beth Israel Deaconess Medical Center, Boston, MA). The pDCR-*ras*(12V) overexpression plasmid encodes active human H-Ras, in which expression is under the control of the cytomegalovirus promoter [49]. The Ras(17N) dominant-negative plasmid inhibits endogenous Ras function [50]. The pRK5-myc-PRAS40 overexpression plasmid encodes wild-type human PRAS40, and was obtained from Do-Hyung Kim (University of Minnesota, Minneapolis, MN) through Addgene [41].

### Transfection and Luciferase Assays

786-0 or Caki-1 (2.5×10^5^ cells) were transfected with the Ras expression plasmids, PRAS40 expression plasmid, or the VEGF promoter-luciferase plasmid using Effectene Transfection Reagent (Qiagen), according to the manufacturer's protocol. The total amount of transfected plasmid DNA was normalized using a control empty expression vector. For luciferase assay, cells were harvested 48 hours after transfection, and luciferase activity was measured using a standard assay kit (Promega) in a luminometer. Transfection efficiency was determined by co-transfection of the β-galactosidase gene under control of cytomegalovirus immediate early promoter and by measurement of β-galactosidase activity using standard assay system (Promega).

### Immunoprecipitation Assays

Immunoprecipitations were performed with 0.5 mg of total protein at antibody excess (1.0 µg/ml) using anti-PRAS40 (Invitrogen). Immunocomplexes were captured with protein A-Sepharose beads (GE Healthcare), and bead-bound proteins were subjected to Western blot analysis using either anti-PKC-ζ or anti-PKC-δ (Santa Cruz Biotechnology).

### Western Blot Analysis

Protein samples were run on SDS-polyacrylamide gel and transferred to a polyvinylidene difluoride membrane (Millipore Corporation). The membranes were incubated with anti-PRAS40 (Invitrogen), anti-phospho-PRAS40 (Invitrogen), anti-Ras (BD Transduction laboratories), anti-VEGF (Santa Cruz Biotechnology), anti-raptor (Cell Signaling) or anti-β-actin (Sigma-Aldrich), and subsequently incubated with peroxidase-linked secondary antibody (Santa Cruz Biotechnology). All primary antibodies were diluted at 0.5 µg/ml; secondary antibodies were diluted at 0.2 µg/ml. The reactive bands were detected by using chemiluminescent substrate (Pierce). Expression was quantified by densitometry using the software Quantity One (version 4.6.2).

### *In Vivo* Tumor Development

Human renal cancer cells (786-0) were injected s.c. in immunodeficient (nu/nu) mice. The tumor volume was measured by following standard method [22], using the formula *V  =  π/6 x a^2^ x b*, wherein *a* is the short axis and *b* is the long tumor axis. Mice were sacrificed at designated times after injection or if complications occurred, which included signs of inactivity, cachexia, or decreased responsiveness. The protocol (# 09-03-1298) for animal studies was approved by the review board of Children's Hospital Boston.

### Immunohistochemistry

Tissue sections were incubated first with either rabbit anti-human phospho-PRAS40 (Invitrogen) or mouse anti-human PRAS40 (Invitrogen), and then with a species-specific horseradish peroxidase-conjugated secondary antibody. Specimens were washed thoroughly in between incubations, developed in 3-aminoethylcarbazole, and counterstained with Gill's hematoxylin.

### Statistical Analysis

Statistical evaluation for data analysis was determined by Student's *t* test. Differences with *P*<0.05 were considered statistically significant.
